# Macrophages and γδ T cells interplay during SARS-CoV-2 variants infection

**DOI:** 10.3389/fimmu.2022.1078741

**Published:** 2022-12-19

**Authors:** Perla Abou Atmeh, Laetitia Gay, Anthony Levasseur, Bernard La Scola, Daniel Olive, Soraya Mezouar, Jean-Pierre Gorvel, Jean-Louis Mege

**Affiliations:** ^1^Aix-Marseille Univ, Institut de Recherche pour le Développement (IRD), Assistance Publique Hopitaux de Marseille (APHM), Microbe Evolution, Phylogeny and Infection (MEPHI), Marseille, France; ^2^Institut Hospitalo-Universitaire (IHU)-Méditerranée Infection, Marseille, France; ^3^Institut Paoli-Calmettes; Aix-Marseille Univ, UM105, Centre National de la Recherche Scientifique (CNRS) UMR 7258, Marseille, France; ^4^Aix-Marseille Univ, Centre National de la Recherche Scientifique (CNRS), Institut National de la Santé et de la Recherche Médicale (INSERM), Centre d’Immunologie de Marseille Luminy (CIML), Marseille, France; ^5^Aix-Marseille Univ, Assitance Publique Hopitaux de Marseille (APHM), Hôpital de la Conception, Laboratoire d’Immunologie, Marseille, France

**Keywords:** SARS-CoV-2 variants, macrophage, ACE2, γδ T cells, COVID-19

## Abstract

**Introduction:**

The emergence of several SARS-CoV-2 variants during the COVID pandemic has revealed the impact of variant diversity on viral infectivity and host immune responses. While antibodies and CD8 T cells are essential to clear viral infection, the protective role of innate immunity including macrophages has been recognized. The aims of our study were to compare the infectivity of different SARS-CoV-2 variants in monocyte-derived macrophages (MDM) and to assess their activation profiles and the role of ACE2 (Angiotensin-converting enzyme 2), the main SARS-CoV-2 receptor. We also studied the ability of macrophages infected to affect other immune cells such as γδ2 T cells, another partner of innate immune response to viral infections.

**Results:**

We showed that the SARS-CoV-2 variants α-B.1.1.7 (United Kingdom), β-B.1.351 (South Africa), γ-P.1 (Brazil), δ-B.1.617 (India) and B.1.1.529 (Omicron), infected MDM without replication, the γ-Brazil variant exhibiting increased infectivity for MDM. No clear polarization profile of SARS-CoV-2 variants-infected MDM was observed. The β-B.1.351 (South Africa) variant induced macrophage activation while B.1.1.529 (Omicron) was rather inhibitory. We observed that SARS-CoV-2 variants modulated ACE2 expression in MDM. In particular, the β-B.1.351 (South Africa) variant induced a higher expression of ACE2, related to MDM activation. Finally, all variants were able to activate γδ2 cells among which γ-P.1 (Brazil) and β-B.1.351 (South Africa) variants were the most efficient.

**Conclusion:**

Our data show that SARS-CoV-2 variants can infect MDM and modulate their activation, which was correlated with the ACE2 expression. They also affect γδ2 T cell activation. The macrophage response to SARS-CoV-2 variants was stereotypical.

## Introduction

Since its emergence in Wuhan (China) in December 2019, severe acute respiratory syndrome coronavirus 2 (SARS‐CoV‐2) caused COVID-19, a pandemic associated with a global health crisis and more than 6.3 million deaths to date (COVID Live - Coronavirus Statistics - Worldometer). SARS-CoV-2 infection may be asymptomatic or can exhibit mild to moderate respiratory disease associating respiratory and digestive symptoms and neurological abnormalities (1, 2). The patients with comorbidities at risk to develop severe illness expressing as acute respiratory distress syndrome characterized by lung injury, inflammation and pulmonary vascular leakage (3, 4). SARS-CoV-2 infection may be also responsible of long-term invalidating symptoms named post-COVID-19 syndrome (5).

The pathogenesis of SARS-CoV-2 infection has been largely imprinted by host immune response (6). Severe COVID-19 patients exhibit a lymphopenia and an impairment of T-cell mediated anti-viral immunity (7). In contrast, few severe patients experience a macrophage activation syndrome (MAS) (8), followed by respiratory and even multi-organ failure (9, 10); and a cytokine release syndrome (CRS) characterized by large amounts of pro-inflammatory cytokines like interleukin (IL)-1, IL-6, IL-8 and tumor necrosis factor (TNF).

Macrophages play a role in the physiopathology of COVID-19 as shown by histological examination of tissue sample from patients with severe symptoms (11, 12). The accumulation of macrophages in the alveolar lumen has been shown in a humanized mice model of SARS-CoV-2 expressing human angiotensin-converting enzyme 2 (ACE2) (13). In addition, post-mortem COVID-19 lung tissue showed an increased proportion of ACE2-positive cells, including a majority of inflammatory macrophages (14, 15). We previously reported that SARS-CoV-2 infects monocyte-derived macrophages (MDM) with abortive infection, similar to SARS-CoV-1 infection (16). According to their function in pathological conditions, macrophages are considered as activated or alternatively activated also referred to as M1 and M2 polarization phenotype, respectively. We showed that SARS-CoV-2 elicited a transcriptional program associating inflammatory and anti-inflammatory genes in macrophages, which shifted to an anti-inflammatory program of M2 type (16). However, there is not a consensus regarding the activation status of macrophages during SARS-CoV-2 infection. Some studies reported a pro-inflammatory response to viruses (17, 18), while others a lack of macrophage activation (19, 20). We still ignore if the activation status of macrophages *in vivo* results from a cytokine-mediated bystander effect or a direct effect of SARS-CoV-2 including its variants.

During the course of COVID-19, the action of the immune system favors SARS-CoV-2 acquiring mutations notably in the virus Spike (S) protein (21). The World Health organization has classified variants into classes: Variants Being Monitored (VBM), Variants of Interest (VOI) and Variants of Concern (VOC). The least hazardous strains are classified as VBM, while VOI are variants that present a possible risk to public health. Finally, VOC are mutated strains of the Wuhan strain that have increased transmissibility, higher disease progression, severity and mortality. In addition, VOC show a decreased susceptibility to vaccine/infection-induced immune responses but they have the ability to reinfect previously infected and recovered individuals. Five SARS-CoV-2 lineages are designated as the VOC: α-B.1.1.7 (United Kingdom), β-B.1.351 (South Africa), γ-P.1 (Brazil), δ-B.1.617 (India) and B.1.1.529 (Omicron) (22, 23).

Here, we investigated the infection and the inflammatory response of MDM in response to Wuhan strain and 5 variants of concern, compared to Vero E6 cell line as the reference model in the study of SARS-CoV-2 infection (24). We also studied the interaction of macrophages infected with other immune cells such as γδ2 T cells, partners of the innate immune response to viral infections. Indeed, previous studies have highlighted the role of γδ2 T cells during SARS-CoV-2 infection (25, 26). We recently demonstrated that activation of γδ2 T cells leads to inhibition of SARS-CoV-2 replication in co-cultures of MDM infected with γδ2 T cells (27). Our data showed that SARS-CoV-2 variants infected MDM and modulated their activation program, which is correlated with ACE2 expression. γδ2 T cell were also found activated. Our study reveals that the macrophages respond to the infection but this one remains stereotypical without specific response against SARS-CoV-2 variants.

## Materials and methods

### Cell culture and infection

Vero E6 (African green monkey kidney, American Type Culture Collection (ATCC^®^ CRL-1586™) cell line was cultured using Minimum Essential Media (MEM, Life Technologies, Carlsbad, CA, USA) supplemented with 10% or 4% fetal bovine serum (FBS, Gibco, Life technologies) and 100 U/mL penicillin and 50 μg/mL streptomycin (Life Technologies).

Blood samples (leucopacks) come from the French Blood Establishment (Etablissement français du sang, EFS) that carries out donor inclusions, informed consent and sample collection. Through a convention established between our laboratory and the EFS (N°7828), buffy coats were obtained and peripheral blood mononuclear cells (PBMC) were isolated as previously described (28). Monocytes were purified from PBMC using anti-CD14-conjugated magnetic beads (Miltenyi Biotec, Bergisch Glabach, Germany) and cultured in Roswell Park Memorial Institute-1640 medium (RPMI, Life Technologies) containing 10% FBS, 2 mM L-glutamine, 100 U/mL penicillin and 50 µg/mL streptomycin. Macrophages derived from monocytes (MDM) were cultured in RPMI-1640 containing 10% inactivated human AB-serum (MP Biomedicals, Solon, OH, USA), 2 mM glutamine, 100 U/mL penicillin and 50 μg/mL streptomycin for 3 days. Then, the medium was replaced by RPMI-1640 containing 10% FBS and 2 mM glutamine, and cells were differentiated into macrophages for 4 additional days.

γδ2 T cells were expanded from fresh PBMCs as previously described (29, 30). Briefly, PBMCs were cultured in RPMI-1640 medium supplemented with 10% FBS, interleukin-2 (IL-2, 200 UI/ml) and Zoledronic acid monohydrate (to a final concentration of 1 µM). IL-2 was added every 2 days beginning on day 5 for 12 days and the purity of the γδ2 T cells was assessed by flow cytometry analysis (>85%) and then frozen at -80°C in 10% dimethyl sulfoxide (Sigma-Aldrich, Saint-Quentin-Fallavier, France) and 90% FBS.

MDM and Vero E6 cells were infected with 20 μl virus suspension at a multiplicity of infection (MOI) of 0.1 for 6, 24, 48 and 72 hours at 37°C in the presence of 5% CO2 and 95% air in a humidified incubator.

### SARS-CoV-2 variant production

SARS-CoV-2 strains, including Wuhan-SARS-CoV-2 (from initial outbreak), α-B.1.1.7 (United Kingdom), β-B.1.351 (South Africa), γ-P.1 (Brazil), δ-B.1.617 (India) and B.1.1.529 (Omicron) was obtained after Vero E6 cells (ATCC^®^ CRL-1586™) infection in MEM supplemented with 4% FBS (31) and virus titration using the median tissue culture infectious dose (TCID50) method. All virus strains were stored at -80°C.

### Viral RNA extraction and q-RTPCR

Viral RNA was extracted using NucleoSpin^®^ Viral RNA Isolation kit (Macherey-Nagel, Hoerdt, France). Virus detection was performed using One-Step RT-PCR SuperScript™ III Platinum™ (Life Technologies). Thermal cycling was achieved at 55°C for 10 minutes for reverse transcription, pursued by 95°C for 3 minutes and then 45 cycles at 95°C for 15 seconds and 58°C for 30 seconds using a LightCycler 480 Real-Time PCR system (Roche, Rotkreuz, Switzerland). We investigated the N gene for the detection of SARS-CoV-2 as previously described (**Table 1**) (32).

**Table 1:** SARS-CoV-2 Nucleocapsid primers and probe.

	Forward primer (5’-3’)	Reverse primer (5’-3’)
**N gene primers**	GACCCCAAAATCAGCGAAAT	TCTGGTTACTGCCAGTTGAATCTG
**N gene probe**	5’ FAM-ACCCCGCATTACGTTTGGTGGACC 3’	

### RNA isolation and q-RTPCR

Total RNA was extracted from MDM (1.10^6^ cells/well) using the RNA extraction Kit (ZYMO Research) with DNase I treatment to eliminate DNA contaminants as previously described (33). The extracted RNAs were evaluated using a NanoDrop spectrophotometer (Nanodrop Technologies, Wilmington, DE, USA). Reverse transcription of isolated RNA was performed using a Moloney murine leukemia virus-reverse transcriptase kit (Life Technologies) and oligo(dT) primers. Real time q-PCR was performed using Smart SYBR Green fast Master kit (Roche Diagnostics, Meylan, France) and specific primers (**Table 2**). Results were normalized using the housekeeping endogenous control *ACTB* gene and were expressed in fold change: 2^-ΔΔCt^ with ΔΔCt = ΔCt_Infected_-ΔCt_Uninfected_.

**Table 2:** List of primers used for q-RTPCR.

Gene	Forward primer (5’-3’)	Reverse primer (5’-3’)
***ACTB***	GGAAATCGTGCGTGACATTA	AGGAGGAAGGCTGGAAGAG
***TNF***	AGGAGAAGAGGCTGAGGAACAAG	GAGGGAGAGAAGCAACTACAGACC
***IL1B***	CAGCACCTCTCAAGCAGAAAAC	GTTGGGCATTGGTGTAGACAAC
***IL6***	CCAGGAGAAGATTCCAAAGATG	GGAAGGTTCAGGTTGTTTTCTG
***TGFB***	GACATCAAAAGATAACCACTC	TCTATGACAAGTTCAAGCAGA
***IL10***	GGGGGTTGAGGTATCAGAGGTAA	GCTCCAAGAGAAAGGCATCTACA
***IFNB***	ACAACCTCCCAGGCACAAGGGCTGTATTT	TGATGGCAACCAGTTCCAGAAGGCTCAAG
***NOS2***	GACTTTCCAAGACACACTTCACC	CTATCTCCTTTGTTACCGCTTCC
***IL1R2***	CACTCAGGTCAGGGCATACTAA	AGGAGAAGAAGAGACACGGATG
***MR***	CTTTCATCACCACACAATCCTC	ACCTCACAAGTATCCACACCATC

### Cell viability

Cell viability was evaluated using the 3-[4,5-dimethylthiazol-2-yl]-2,5 diphenyl tetrazolium bromide (MTT) assay. After a 24, 48 and 72 hours of SARS-CoV-2 stimulation, 10 μl of MTT (5 mg/ml, Sigma-Aldrich) were added to the cell cultures and incubated at 37°C for 4 hours. The formed formazan crystals were solubilized with 50 μl of dimethylsulphoxide (DMSO) for 30 minutes at 37°C and quantified using a Synergy MxF plate reader at 540 nm (Biotek Instruments, Winooski, VT, USA).

### Immunofluorescence

MDM and Vero E6 cells (5.10^5^ cells/well) cultured into a 24-well plate containing a glass coverslip were fixed with 4% paraformaldehyde at 4°C for 20 minutes and then permeabilized with 0.1% Triton X-100 in phosphate-buffered saline (PBS) for 3 minutes. Permeabilized cells were incubated with blocking buffer (3% bovine serum albumin diluted in PBS) for 30 minutes and then with primary SARS/SARS-CoV-2 Coronavirus Spike Protein (subunit 1) (1:250, Life Technologies) and ACE2 (1:250, R&D systems, Minneapolis, MN, USA) antibodies for 1 hour. Coverslips were then washed three times with PBS and incubated for 30 minutes at room temperature with secondary antibodies: anti-rabbit Alexa Fluor 633 and anti-mouse Alexa Fluor 488 (1:1000, Invitrogen). Phalloïdin-647 (1:250) and 4’,6-diamidino-2-phenylindole (DAPI, 1:250) were also added to reveal F-actin and nuclei, respectively. An LSM800 Airyscan confocal microscope (Zeiss, Germany) with a 63x oil objective was used. Relative ACE2 expression was quantified by fluorescence with ImageJ software (National Institutes of Health, Bethesda, MD, USA). Relative percentage of ACE2 fluorescence was reported to DAPI fluorescence.

### Immunoassays

Cytokine release was evaluated from supernatants of infected MDMs at 24 and 48 hours post-infection. Tumor necrosis factor TNF-α, interleukin IL-10, IL-1β (R&D Systems), and IL-6 (CliniSciences, Montrouge, France) were quantified according to the manufacturer’s recommendations. The sensitivity was (pg/ml): 5.5 for TNF-α, 3.9 for IL-10, 0.125 for IL-1β, and 15.4 for IL-6.

### Flow cytometry

Cells (1.10^6^ cells/well) were suspended in PBS containing 5% FBS and 2mM EDTA (Sigma-Aldrich). Suspended cells were incubated with viability dye (Live/Dead Near IR, Invitrogen), CD14-FITC, anti-ACE-2-PE or appropriate isotype control (Miltenyi) for 30 minutes at 4°C. Labelled cells were then permeabilized using BD Cytofix/Cytoperm kit and stained with CD68-PE-Cy7 (Miltenyi). Data were collected on a Navios instrument (Beckman Coulter) and analyzed with FlowJo software (FlowJo v10.6.2, Ashland, OR).

### γδ2 T cells activity

MDM were infected for 24 hours with the different SARS-CoV-2 variants studied at an MOI of 0.1. The MDMs were then co-cultured with γδ2 T cells at effector-to-target (E:T) ratio of 1:1 in presence of GolgiStop (BD Biosciences) and CD107(a+b)-FITC (BD Biosciences). Phorbol 12-myristate 13-acetate (PMA, 20 ng/mL) with ionomycine (1 µg/mL) was used as positive control for γδ2 T cell activation. After 4 hours, cells were harvested and stained with a viability marker (Live/Dead Near IR), CD3-PE-Cy7 and TCRγ/δ-PE (Miltenyi Biotec). Fixation/permeabilization kit (BD Biosciences) was used for intracellular staining with TNFα-eFluor 450 and IFNγ-APC (eBioscience). Data were collected on a Navios instrument (Beckman Coulter) and analyzed with FlowJo software (FlowJo v10.6.2).

### Statistical analysis

Statistical analysis was performed with GraphPad Prism (7.0, La Jolla, CA), using the two-way ANOVA test. Transcriptional data were analyzed using the ClustVis webtool. Significance was set at *p*<0.05.

## Results

### Macrophage infection with SARS-CoV-2 variants

**Figure 1:**
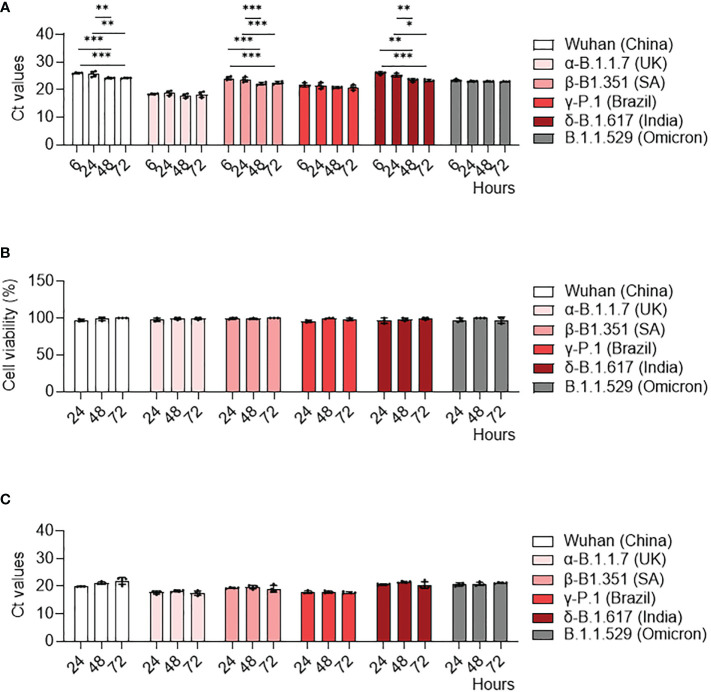
MDM infection with SARS-CoV-2 variants. MDM were infected with SARS-CoV-2 variants including Wuhan (China), α-B.1.1.7 (United Kingdom, UK), β-B.1.351 (South Africa, SA), γ-P.1 (Brazil), δ-B.1.617 (India) and B.1.1.529 (Omicron) (0.1 MOI) for 6, 24, 48 and 72 hours. **(A)** SARS-CoV-2 was quantified in cells as Ct values by RT-PCR. **(B)** Cell viability was tested at 24, 48 and 72 hours post-infection. **(C)** SARS-CoV-2 replication was quantified by RT-PCR in cell supernatants and expressed as Ct values. Data values represent the mean ± SD from 4 healthy donors whose experiments were carried out in triplicate. Statistical analysis was performed with two-way ANOVA and Tukey’s multiple comparison test. ^*^p ≤ 0.05, ^**^p ≤0.01 and ^***^p ≤ 0.001.

We previously showed infection properties of MDM using the Wuhan (china) strain (34). We then wondered if SARS-CoV-2 variants exhibited a similar response in MDM. We infected MDM with viruses at 0.1 MOI for 6, 24, 48 and 72 hours and we measured their infection rate with qRT-PCT. We showed that MDM were infected with all variants (**Figure 1**). Furthermore, the γ-P.1 (Brazil) variant was more efficient at infecting MDM than the other variants (**Figure 1A**), something that was not detected in infected Vero E6 cells (**Supplementary Figure 1A**), the reference cell model for the study of SARS-CoV-2 (35). In addition, we reported a significant increase in viral load at 24, 48 and 72 hours post-infection compared to the Wuhan (china), δ-B.1.617 (India) and β-B.1.351 South African variants at 6 hours post-infection (**Figure 1A**). In contrast, all SARS-CoV-2 variants led to a strong increase in viral load in Vero E6 cells (**Supplementary Figure 1A**). Thus, despite small variations, SARS-CoV-2 variants did not efficiently replicate in MDM.

Then, we studied how can SARS-CoV-2 variants induce a cytopathic effect as assessed by cell viability assay. No cytopathic effect was observed in MDM infected by any of the SARS-CoV-2 variants (**Figure 1B**). In contrast, all SARS-CoV-2 variants induced a cytopathic effect in Vero E6 cells, with a 70% mortality at 72 hours post-infection (**Supplementary Figure 1B**).

Finally, we quantified SARS-CoV-2 viral load in MDM supernatants to study viral replication. In contrast to Vero E6 cells (**Supplementary Figure 1C**), the viral load of all SARS-CoV-2 variants did not change over time (**Figure 1C**). Taken together, despite the higher infectivity of the γ-Brazil variant, SARS-CoV-2 variants shared the ability to infect MDM without replication.

### Macrophage inflammatory response to SARS-CoV-2 variants

**Figure 2:**
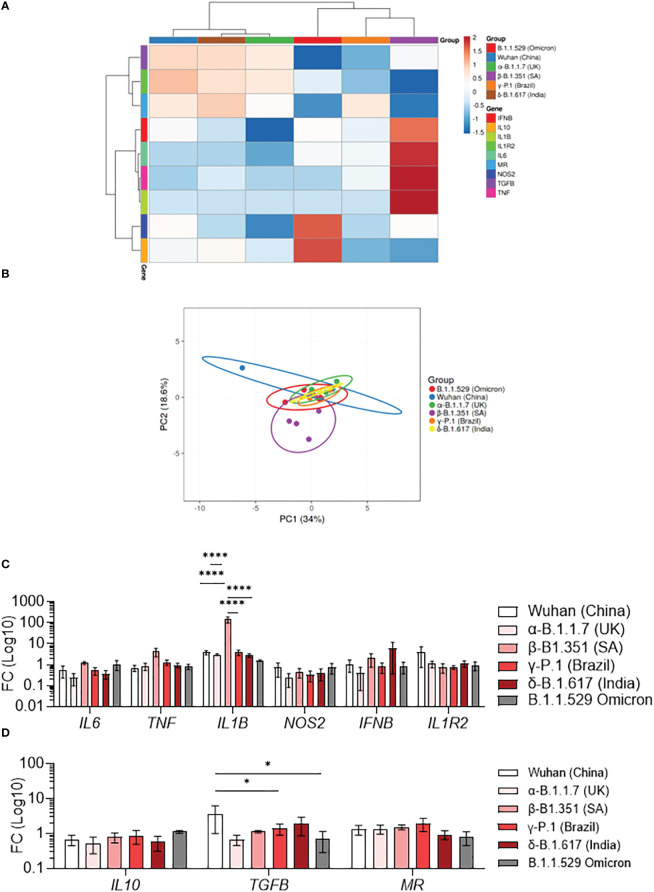
Polarization profile of MDM infected with SARS-CoV-2 variants. MDMs were infected with SARS-CoV-2 variants including Wuhan (China), α-B.1.1.7 (United Kingdom, UK), β-B.1.351 (South Africa, SA), γ-P.1 (Brazil), δ-B.1.617 (India) and B.1.1.529 (Omicron) (0.1 MOI). **(A–D)** The polarization status was investigated by measuring the expression of M1 genes (*IL6*, *TNF*, *IL1B*, *NOS2*, *IFNB, IL1R2*) and M2 genes (*IL10*, *TGFB*, *MR*) at 6 hours post-infection. Data are illustrated as **(A)** hierarchical clustering and **(B)** principal component analysis obtained using ClustVis webtool. **(C, D)** Fold change (FC) of **(C)** M1 genes and **(D)** M2 genes (Log 10). Data values represent the mean ± SEM from 4 healthy donors whose experiments were carried out in triplicate. Statistical analysis was performed with one-way ANOVA and Tukey’s multiple comparison test. ^*^p ≤ 0.05 and ^****^p ≤ 0.0001.

Macrophages activation is usually classified in M1 category (pro-inflammatory) and M2 category (anti-inflammatory). We have previously shown that SARS-CoV-2 induces a specific reprogramming of MDM towards an atypical M2 polarization (34). Therefore, we wondered if infection with different SARS-CoV-2 variants could affect the MDM polarization program. We measured the expression of 6 M1-related genes (*IL6, TNF, IL1B, NOS2, IFNB, IL1R2*) and 3 M2-related genes (*IL10, TGFB, MR*) by q-RTPCR in MDM infected with SARS-CoV-2 variants. First, the hierarchical clustering showed two clusters of infected MDM (36): Wuhan, α-B.1.1.7 (United Kingdom) and δ-B.1.617 (India) (1) γ-P.1 (Brazil), β-B.1.351 South Africa, and B.1.1.529 (Omicron) variants (**Figure 2A**). Interestingly, the principal component analysis of gene expression showed that MDM infected with the β-B.1.351 (South Africa) variant formed a distinct group from the other SARS-CoV-2 variants (**Figure 2B**). The hierarchical clustering revealed a tendency of increased expression of M1-related genes i.e. *TNF*, *IL1B* and *IL6* in MDM infected with the β-B.1.351 (South Africa) variant compared to the other SARS-CoV-2 variants (**Figure 2A**). However, only the expression of *IL1B* gene was significantly increased in MDM infected with the β-B.1.351 (South Africa) variant compared to the other variants (*p<0.0001*) (**Figure 2C**). On the other hand, for M2-related gene expression, a significant increase in *TGFB* expression was found for the Wuhan SARS-CoV-2 compared to the γ-P.1 (Brazil) and B.1.1.529 Omicron variants (*p<0.05*). The expression of *IL10* and *MR* was not modulated upon infection with all variants (**Figure 2D**).

**Figure 3:**
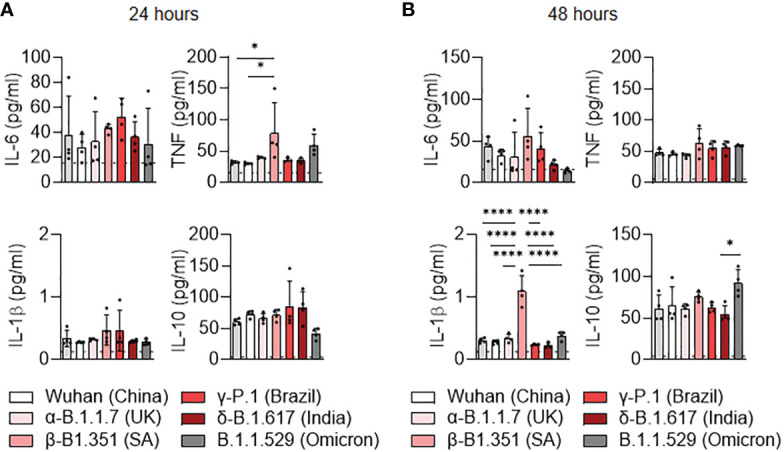
Cytokine release of MDM infected with SARS-CoV-2 variants. **(A, B)** Levels of TNF, IL-6, IL-10 and IL1-β were evaluated in the culture supernatants by ELISA at **(A)** 24 and **(B)** 48 hours post-infection. Data values represent the mean ± SEM from 4 healthy donors whose experiments were carried out in triplicate. Statistical analysis was performed with one-way ANOVA and Tukey’s multiple comparison test. ^*^p ≤ 0.05 and ^****^p ≤ 0.0001.

We then investigated the cytokine secretion induced by SARS-CoV-2 variants at 24 hours (**Figure 3A**) and 48 hours (**Figure 3B**) post-infection. The TNF production was significantly increased at 24 hours post-infection in β-B.1.351 (South Africa) infected-MDM compared to uninfected MDM and Wuhan-infected MDM (*p=0.0279*) (**Figure 3A**). IL-1β over production was also observed in β-B.1.351 (South Africa) infected-MDM at 24h post-infection in comparison to the other SARS-CoV-2 variants and uninfected MDM (all *p<0.0001*) (**Figure 3B**), respectively. We noticed a significant increase in IL-10 secretion in B.1.1.529 (Omicron) infected MDM compared to infections with the δ-B.1.617 (India) variants (*p=0.0244*). Overall, these results do not illustrate a polarization of MDM. Nevertheless, unlike the other variants, the β-B.1.351 (South Africa) variant induced macrophage activation while the B.1.1.529 (Omicron) was rather inhibitory.

### Modulation of ACE2 expression by macrophages infected with SARS-CoV-2 variants

**Figure 4:**
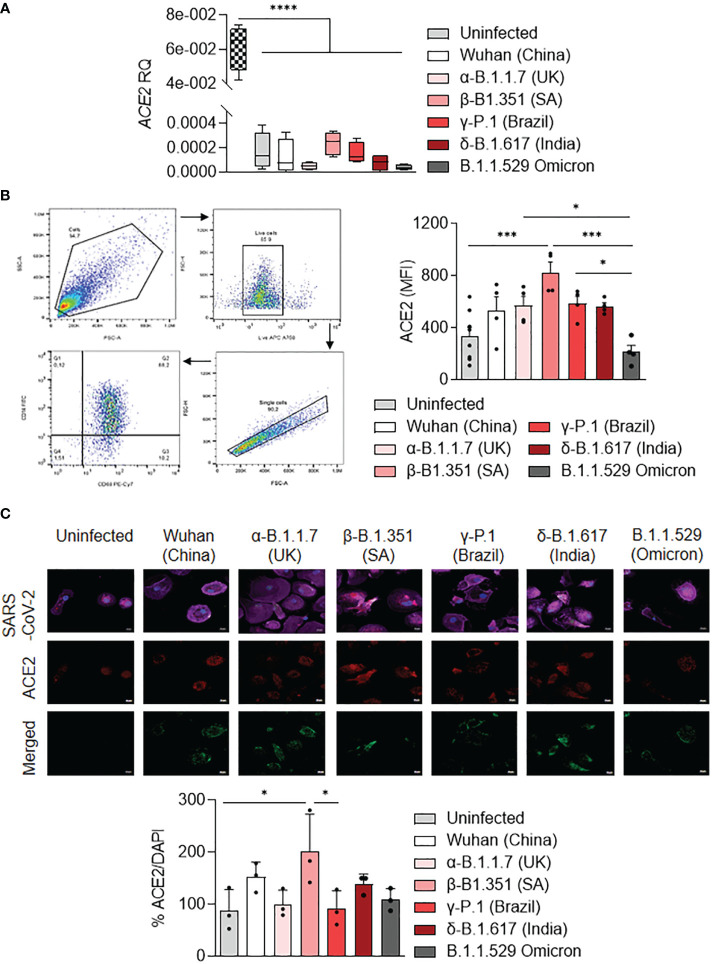
ACE2 expression by MDM infected with SARS-CoV-2 variants. MDMs were infected with SARS-CoV-2 variants including Wuhan (China), α-B.1.1.7 (United Kingdom, UK), β-B.1.351 (South Africa, SA), γ-P.1 (Brazil), δ-B.1.617 (India) and B.1.1.529 (Omicron) (0.1 MOI). **(A)** Relative quantity of *ACE2* gene was evaluated by q-RTPCR at 6 hours post-infection after normalization with housekeeping *ACTB* gene as endogenous control. Data values represent the mean ± SD from 4 healthy donors, and the experiments on unstimulated Vero E6 cells were performed in triplicate. **(B)** ACE2 protein expression was quantified by flow cytometry in MDMs at 24 hours post-infection and expressed as mean fluorescence intensity (MFI) values. **(C)** ACE2 was evaluated by immunofluorescence in MDMs at 24 hours post-infection. ACE2 was identified in red, SARS-CoV-2 in green, F-actin in purple and DAPI. Relative ACE2 expression was quantified by fluorescence with ImageJ software. Statistical analysis was performed with two-way ANOVA and Tukey’s multiple comparison test. ^*^p ≤ 0.05, ^***^p ≤ 0.001 and ^****^p ≤ 0.0001.

It has been shown that ACE2 expression was higher on the LPS-activated M1 macrophages compared to IL-4-treated M2 macrophages (37). Thus, we tested the activation profile in stimulated MDM infected with SARS-CoV-2 variants in relationship to ACE2 expression. We showed that *ACE2* gene expression was lower in uninfected or stimulated MDM compared to unstimulated Vero E6 cells (*p<0.0001*). *ACE2* gene expression was higher in MDM infected with the β-B.1.351 (South Africa) variant and lower in MDM infected with the α-B.1.1.7 (United Kingdom) and B.1.1.529 (Omicron) variants (**Figure 4A**). The ACE2 protein expression was quantified by flow cytometry and immunofluorescence in infected MDM. We reported that the ACE2 protein expression was higher in MDM infected with the β-B.1.351 (South Africa) variant compared to the other variants (B.1.1.529 (Omicron), *p=0.0002*) and uninfected cells (*p=0.0002*). In contrast, MDM infected with the B.1.1.529 (Omicron) presented the lowest levels of ACE2 expression (α-B.1.1.7 (United Kingdom), *P=0.0472* and γ-P.1 (Brazil) variants, *p=0.0355*) (**Figure 4B**). This high ACE2 expression for the South Africa variant was also found by immunofluorescence analysis. Indeed, an increase of ACE2 expression was observed in MDM infected with the β-B.1.351 (South Africa) variant compared to the other variants (γ-P.1 (Brazil), *p<0.05*) and uninfected cells (*p<0.05*) (**Figure 4C**).

Taken together, the results showed that SARS-CoV-2 variants modulate ACE2 expression in MDM. In particular, the β-B.1.351 (South Africa) variant induced a higher expression of ACE2, in relationship to MDM activation.

### Macrophages infected with SARS-CoV-2 variants induce a different activation of γδ2 T cells

**Figure 5:**
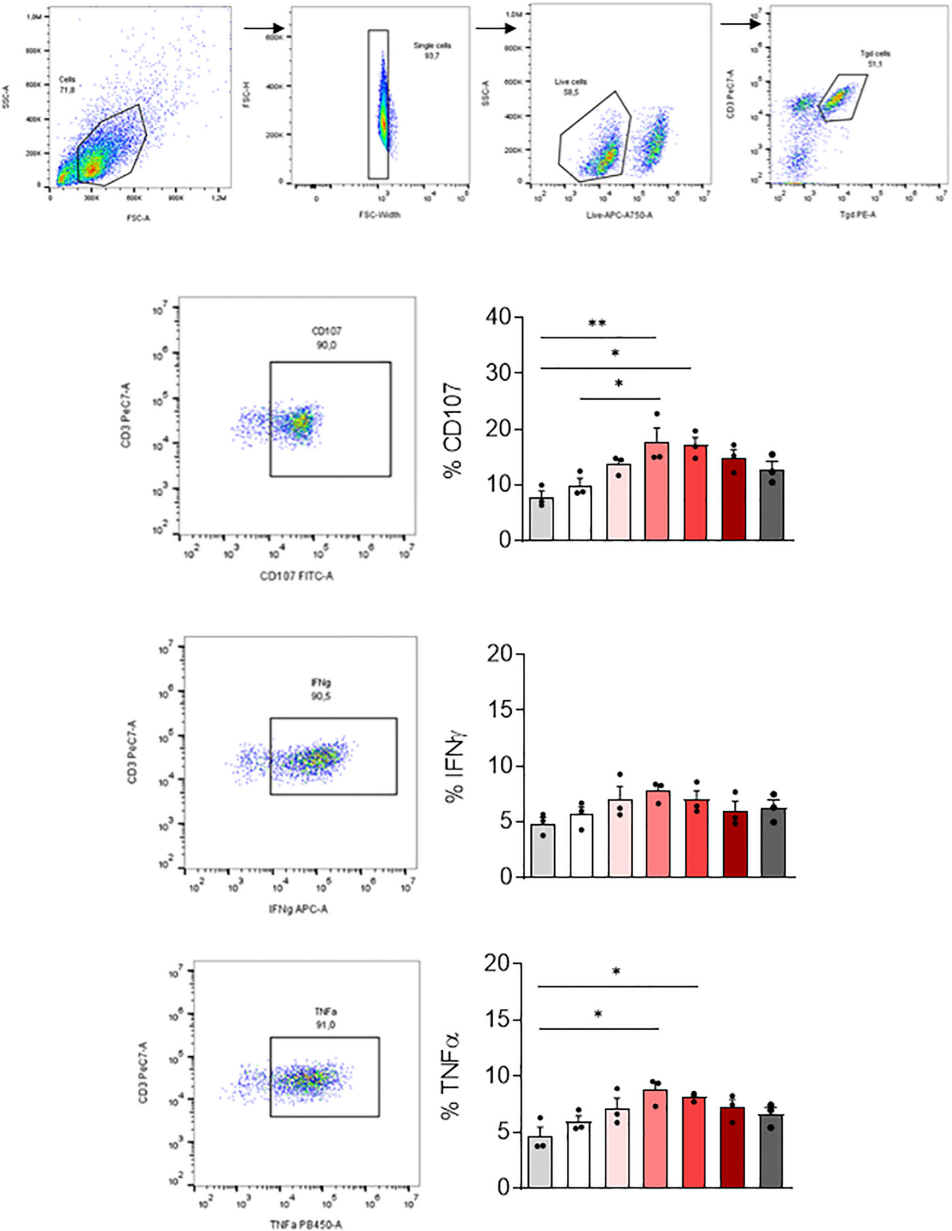
γδ2 T cell activation. MDM previously infected 24 hours with SARS-CoV-2 variants including Wuhan (China), α-B.1.1.7 (United Kingdom, UK), β-B.1.351 (South Africa, SA), γ-P.1 (Brazil), δ-B.1.617 (India) and B.1.1.529 (Omicron) (0.1 MOI) were co-cultured with autologous γδ2 T cells (E:T ratio of 1:1). γδ2 T cell degranulation (% CD107ab^+^ cells) and intracellular TNFα and IFNγ, respectively, were assessed after 4 hours of co-culture in the presence of GolgiStop and analyzed by flow cytometry. Manual gating to identify γδ2 T cell population (CD3^+^ TCRVδ2^+^). The percentage of CD107^+^, IFNγ^+^ and TNFα^+^ cells, were then gated in the γδ2 cell population (CD3^+^ TCRVδ2^+^). Data values represent the mean ± SD from 3 healthy donors. Statistical analysis was performed with one-way ANOVA and Tukey’s multiple comparison test. ^*^p ≤ 0.05 and ^**^p ≤0.01.

We then tested if MDM infected with SARS-CoV-2 variants affected differently the activation of other immune cells playing an important role in COVID-19. For this purpose, we co-cultured SARS-CoV-2 variants infected-MDM with autologous γδ2 T lymphocytes, which play a role during SARS-CoV-2 infection (25, 27). γδ2 T lymphocytes kill infected cells by direct cytotoxicity through the secretion of cytolytic molecules (perforin and granzymes) and by a cell-mediated non-cytolytic activity based on cytokine production (IFN-γ and TNF-α secretion) (38). Therefore, we assessed γδ2 T cell degranulation (% CD107ab^+^ cells) by flow cytometry (**Figure 5**). We showed that MDM infected with the γ-P.1 (Brazil) and β-B.1.351 (South Africa) variants induced a higher degranulation of γδ2 T cells than unstimulated MDM (*p=0.0108* and *p=0.0071*, respectively) (**Figure 5**).

Since γδ2 T cells exert their antiviral activity in a cytokine-dependent manner, we analyzed their TNF-α and IFN-γ production by flow cytometry. Infection of MDM with the γ-P.1 (Brazil) and β-B.1.351 (South Africa) variants resulted in highest TNF-α secretion by γδ2 T cells (*p=0.0324* and *p=0.0101*, respectively).

In summary, all variants were able to activate γδ2 cells. γ-P.1 (Brazil) and β-B.1.351 (South Africa) variants were the most efficient.

## Discussion

In this study, we analyzed how SARS-CoV-2 variants differently infect human macrophages and modulate their function. We compared SARS-CoV-2 variant infection using two cell models, Vero E6, African green monkey kidney cell line, which is widely used for SARS-CoV-2 isolation and virus production, as a positive control (39, 40) and MDM, a model of tissue macrophage of hematopoietic origin (41). Firstly, we showed that Vero E6 were more infectable than macrophages, likely related to abundant expression of ACE2 by Vero E6 cells. This was in accordance with previous study in which Vero E6 are more permissive for SARS-CoV-2 infection than primary cells (42). Secondly, we reported that all SARS-CoV-2 variants were able to infect macrophages, the γ-P.1 (Brazil) variant being most efficient as compared to the other variants. In contrast, we did not find any difference in infectibility among the SARS-CoV-2 variants in Vero E6 cells. This may indicate that internalization of viruses cannot be a means to predict the severity of the disease.

The interaction of viruses like SARS-CoV-2 with macrophages induces their activation, which may lead to tissue damage and severe disease *via* the production of inflammatory and toxic mediators (9). As we previously demonstrated, macrophage activation can be stratified into M1 and M2 states using a combination of markers (11, 43, 44). It was recently reported that polarized M1 and M2 macrophages presented an inhibitory effects on SARS-CoV-2 infection (45). More interestingly the authors showed that, in contrast to M2 macrophages, M1 and un-activated M0 macrophages up-regulated inflammatory factors. Here, we wondered whether infection with the different variants could lead to a distinct immune response in macrophages and contribute to the observed clinical differences. Our results show that infection led to the expression of genes associated with either M1 or M2 profile, suggesting that SARS-CoV-2 does not induce a clear macrophage polarization. This is consistent with a previous study, where we showed that the α-SARS-CoV-2 (Wuhan) variant induced an early M1/M2 followed by a late M2 program in macrophages (16). More specifically, macrophages infected with the β-B.1.351 (South Africa) variant showed a transcriptional program characterized by the up-regulation of M1-type genes validated by an increased secretion of TNF and IL-1β. Although the number of modulated macrophage markers was small, it seems that the β-B.1.351 (South Africa) variant was more efficient than the other variants to reprogram macrophages toward an M1 profile. In contrast, the Omicron variant seems to be less able to polarize macrophages toward an M1 profile *via* its ability to induce IL-10 secretion. These finding highlight the concept of targeting macrophage in COVID-19 as a current and future therapeutic strategy as it was reported that blocking macrophage pro-inflammatory molecules such as the treatment by IL-1α/β inhibitor anakinra provided encouraging perspectives (46).

The viral load in SARS-CoV-2 infected macrophages remained unchanged during the time of the culture. This was emphasized by the lack of cytopathic effects in response to all variants. This shows that infection with all SARS-CoV-2 variants in macrophages presents no replication although a discrete increased viral load was observed with α-SARS-CoV-2 (Wuhan), δ-B.1.617 (India) and β-B.1.351 (South Africa) variants. This is reminiscent of previous studies showing that SARS-CoV-2 efficiently infects human macrophages without replication (17, 47, 48), similar to SARS-CoV-1 (16, 49–51) suggesting a protective role for macrophage during SARS-CoV infection at it was recently reported in humanized mice model (52). It is likely that variations in macrophage response to SARS-CoV-2 variants may be a consequence of changes in ACE2 expression. Several recent studies reported a link between macrophage polarization and ACE2 expression (37, 45, 53). Indeed, ACE2 expression has been shown to be higher in LPS-activated M1 macrophages than in IL-4-treated M2 macrophages (37). The inhibition of viral entry using ACE2 blocking antibody enhances the activity of M2 iPSC-derived macrophages (45). Therefore, we investigated the ACE2 expression in macrophages infected with the different variants. It is noteworthy that the expression of ACE2 by macrophages was markedly lower than that of Vero E6 cells. Interestingly, among the response to different variants of concern, β-B.1.351 (South Africa) infected macrophages expressed higher levels of ACE2 at the cell surface than uninfected macrophages or B.1.1.529 (Omicron) infected macrophages. These results suggest that elevated levels of pro-inflammatory cytokines increase ACE2 expression in an autocrine manner, facilitating cell infectivity. In the case of the β-B.1.351 (South Africa) variant, the M1 profile may induce a higher ACE2 expression, and explains the increased infectivity of macrophages during the infection kinetics. In contrast, IL-10 overproduced in response to B.1.1.529 (Omicron) may decrease ACE2 expression and limit virus-mediated inflammatory response.

Finally, macrophages infected with the variants displayed a different effect on γδ2 T cells whose antiviral properties are promoted by macrophages (54, 55). We recently reported that activated γδ2 T cells elicit *in vitro* strong cytotoxic and non-cytolytic anti-SARS-CoV-2 activities in response to the Wuhan strain (27). Using an *in vitro* co-culture model, we studied SARS-CoV-2 variant-infected macrophage impact on the activation of γδ2 T cells in response to each variant of concern. We revealed that infection of MDM with the γ-P.1 (Brazil) and β-B.1.351 (South Africa) variants resulted in higher γδ2 T cell activation.

Our results suggest that the β-B.1.351 (South Africa) variant possesses molecular characteristics that account for its specific impact on macrophages. β-B.1.351 (South Africa) variant is known to be less sensitive to neutralizing antibodies (56) and to exhibit increased affinity for ACE2 compared with Wuhan receptor binding domain (RBD). This latter is the result of the triple mutation K417N, E484R, and N501Y that is characteristic of the β-B.1.351 (South Africa) RBD. Therefore, antibodies of lower affinity will struggle to compete with ACE2 receptor (57). These facts may explain why we observed an upregulation in ACE2 expression in macrophages infected with the β-B.1.351 (South Africa) variant, suggesting that escaping from neutralizing antibodies may enhance the activation of macrophages and innate immunity in an ACE2 receptor-dependent manner.

We have previously reported the importance of several models to investigate SARS-CoV-2 infection *in vitro* and *in vivo* (24). Vero E6 cell line constitutes a reference model in the study of SARS-CoV-2 infection due to an abundant expression of ACE2 receptor on their membrane (58, 59). Nevertheless, Vero E6 presents several disadvantages, such as low expression of *IFN* genes (60, 61) and the absence of TMPRSS2, an essential protein for SARS-CoV-2 viral entry (58). In the absence of TMPRSS2, SARS-CoV-2 may be proteolytically activated following receptor-mediated endocytosis by cathepsin B/L (58, 62). To investigate the role of macrophage in SARS-CoV-2 infection we used the MDM model that it was previously reported to express both ACE2 and TMPRSS2 proteins (37, 63) constituting a more relevant model to the actual disease in humans.

In conclusion, SARS-CoV-2 variants modulate both macrophage activation program including γδ2 T cells and ACE2 expression. Among the variants of concern, the β-B.1.351 (South Africa) variant is highlighted thanks to its efficacy to induce an M1-related program, γδ2 T cell activation and ACE2 overexpression. The characteristics of β-B.1.351 (South Africa) mutations may explain this specific effect on macrophages although the molecular impact of these mutations has still to be clearly deciphered. On the other hand, the Omicron variant is the only one able to stimulate IL-10, known for its immunoregulatory properties, which may account for its decreased pathogenicity. This study demonstrates that the diversity of SARS-CoV-2 has an impact on macrophages and this must be taken into account to understand the immunopathology of COVID-19 and the treatment of patients with new therapies such as cytokine antagonists or antibody targeting virus receptors.

## Data availability statement

The original contributions presented in the study are included in the article/**Supplementary Material**. Further inquiries can be directed to the corresponding author.

## Ethics statement

Peripheral blood mononuclear cells (PBMC) were isolated as previously described (27) from deidentified blood samples (leucopacks) come from the French Blood Establishment (Etablissement français du sang, EFS) that carries out donor inclusions, informed consent and sample collection. Through a convention established between our laboratory and the EFS (N°7828), buffy coats were obtained and monocytes were isolated for this study. The patients/participants provided their written informed consent to participate in this study.

## Author contributions

PA and LG performed the experiments, analyzed the data; PA and SM wrote the first draft of the manuscript; AL performed the bioinformatics experiment and analysis; BL provided SARS-CoV-2 strains; DO, SM, J-PG, and J-LM supervised the work and wrote the final manuscript. All authors contributed to the article and approved the submitted version.

## Supplementary material

Supplementary Figure 1 Vero E6 cell line infection with SARS-CoV-2 variants Vero E6 cells were infected with SARS-CoV-2 variants including Wuhan (China), α-B.1.1.7 (United Kingdom, UK), β-B.1.351 (South Africa, SA), γ-P.1 (Brazil), δ-B.1.617 (India) and B.1.1.529 (Omicron) (0.1 MOI) for 6, 24, 48 or 72 hours. **(A)** Cell viability was tested at 24, 48 and 72 hours post-infection. **(B)** The presence of SARS-CoV-2 was evaluated and quantified by immunofluorescence (left panel, virus in green, nucleus in blue and F-actin in purple) and RT-PCR expressed as Ct values (right panel), respectively. **(C)** SARS-CoV-2 replication was quantified by RT-PCR in cell supernatant and expressed as Ct values. Data values represent the mean ± SD from three independent experiments in triplicate. Statistical analysis was performed with two-way ANOVA and Tukey’s multiple comparison test. ^*^*p ≤ 0.05*, ^**^*p ≤0.01*, ^***^*p ≤ 0.001* and ^****^*p ≤ 0.0001*.
