# Supplementary figures and images for: Macrophages and γδ T cells interplay during SARS-CoV-2 variants infection
Source: Front Immunol. 2022 Dec 19;13:1078741. doi: 10.3389/fimmu.2022.1078741 (PMC9806226; doi:10.3389/fimmu.2022.1078741)

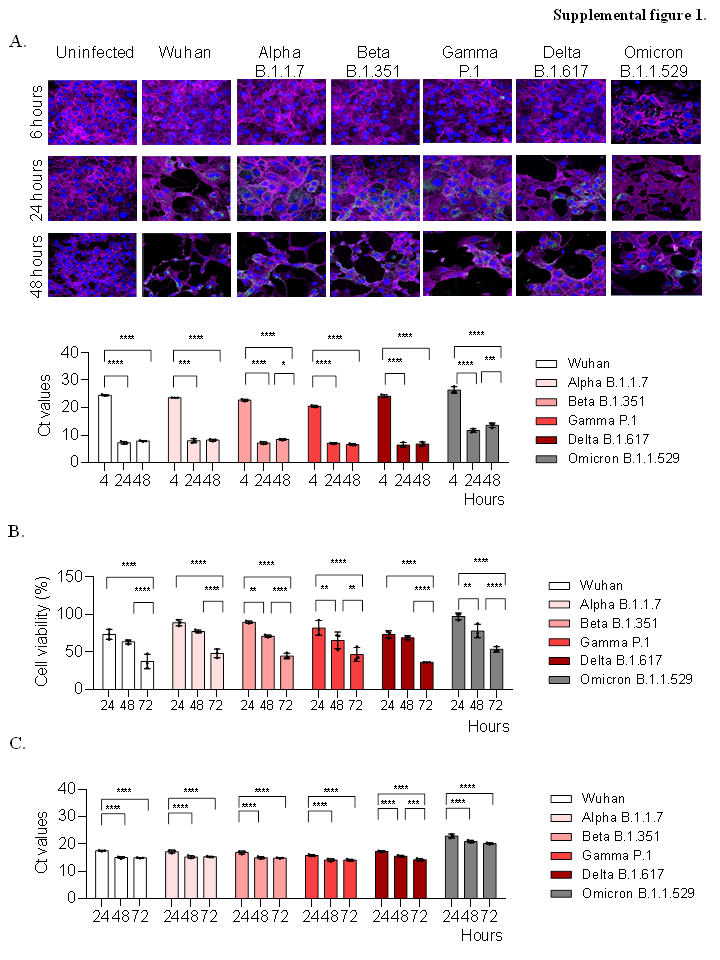

Supplement: Supplementary Figure 1 — Vero E6 cell line infection with SARS-CoV-2 variants Vero E6 cells were infected with SARS-CoV-2 variants including Wuhan (China), α-B.1.1.7 (United Kingdom, UK), β-B.1.351 (South Africa, SA), γ-P.1 (Brazil), δ-B.1.617 (India) and B.1.1.529 (Omicron) (0.1 MOI) for 6, 24, 48 or 72 hours. (A) Cell viability was tested at 24, 48 and 72 hours post-infection. (B) The presence of SARS-CoV-2 was evaluated and quantified by immunofluorescence (left panel, virus in green, nucleus in blue and F-actin in purple) and RT-PCR expressed as Ct values (right panel), respectively. (C) SARS-CoV-2 replication was quantified by RT-PCR in cell supernatant and expressed as Ct values. Data values represent the mean ± SD from three independent experiments in triplicate. Statistical analysis was performed with two-way ANOVA and Tukey’s multiple comparison test. *p ≤ 0.05, **p ≤0.01, ***p ≤ 0.001 and ****p ≤ 0.0001. [file Image_1.tif]
